# Insights Into ‘Living Flat’: A Qualitative Study of Patients Who Have Mastectomy Without Reconstruction

**DOI:** 10.1002/pon.70436

**Published:** 2026-03-26

**Authors:** Laura Lyons, Charlene Martin, Lynda Wyld, Nicola King, Sam Brunsden, Jenna Morgan

**Affiliations:** ^1^ Division of Clinical Medicine University of Sheffield Medical School Sheffield UK; ^2^ Doncaster and Bassetlaw Teaching Hospitals NHS Trust Jasmine Center Doncaster Royal Infirmary Doncaster UK; ^3^ Flat Friends UK London UK

**Keywords:** breast cancer, mastectomy, shared decision making, surgery

## Abstract

**Background:**

Over 14,000 UK women undergo mastectomy annually, 70% of whom do not undergo breast reconstruction. These women are often left with suboptimal scars.

**Aims:**

This study aimed to explore the attitudes of women towards their mastectomy scars, particular the aesthetic outcomes, and to raise awareness of the importance of achieving aesthetic flat closure.

**Methods:**

Semi‐structured interviews with women who had undergone mastectomy without reconstruction were recruited from a single UK teaching hospital. Thematic analysis was performed using the Framework Approach.

**Results:**

Twenty women aged 47–91 years old were interviewed in 2024. Themes identified: satisfaction with scar cosmesis, physical impacts of scars, attitude towards flat mastectomy scars, body image and confidence, pre‐operative counselling and scar revision surgery. There was widespread patient dissatisfaction with mastectomy scar cosmesis, although attitudes and emotional responses varied. Women viewed their scars as a necessity. Physical symptoms included discomfort from dog ears chafing on bra straps, or scar tightness restricting arm or shoulder mobility. Most women were interested in scar revision surgery, with some requesting more information, or referrals. Those who had scar revision surgery or chose contralateral symmetrising mastectomy displayed more positive attitudes.

**Conclusions:**

This study found considerable patient dissatisfaction with mastectomy scar cosmesis and demonstrated the profound impacts of poor cosmetic outcomes, highlighting the importance of optimising aesthetic outcomes and the need for scar revision surgery to help women achieve aesthetic flat closure. These findings demonstrate the importance of careful pre‐operative planning and good surgical technique with more time allocated to manage expectations.

## 1 Background

As breast cancer survival rates increase, more women are living with the after‐effects of breast cancer surgery, meaning the outcomes and impacts of breast cancer surgery are increasingly important [1]. In the UK, 70% of the estimated 14,850 women who undergo mastectomy annually, will not undergo breast reconstruction [2, 3]. This is often referred to as ‘going flat’. The primary reason patients undergo mastectomy is treatment of breast cancer, where they are unsuitable for, or as an alternative to, breast conserving surgery. However, some may also undergo mastectomy to reduce their risk of developing breast cancer (due to a genetic abnormality conferring increased risk) ‐ usually in the form of bilateral risk‐reducing mastectomy. Breast reconstruction (either autologous or implant‐based) may be offered to patients to recreate the breast mound but is associated with additional surgical time, risks and side‐effects. Some patients may opt to undergo contralateral symmetrising mastectomy (CSM) following cancer treatment. This involves the removal of the contralateral healthy breast to achieve flat symmetry, not to reduce breast cancer risk.

There are many reasons why women have a mastectomy without reconstruction (MxNR): cancer factors, lack of information on surgical options, being an unsuitable candidate for reconstruction, or it may be patient choice [4, 5, 6]; with some women preferring to ‘go flat’ or wishing to avoid further or more complex surgeries and shorten recovery time [7]. For those who don't have reconstruction, the cosmesis and function of their MxNR remains important. There is increasing recognition of the importance of aesthetic flat closure which involves intentional incision selection and manoeuvres to refine the contour of the skin flaps to optimize the cosmetic result of an MxNR [8, 9, 10]. These include techniques to minimise excess tissue and skin at the end of the scars (often colloquially known as ‘dog ears’). Such incisions include extending the MxNR scar laterally, curving the lateral portion of the incision to include lateral excess and specific ‘fish‐tail plasty’ or ‘Fleur de Lis’ type incisions.

Unfortunately, women are often left with poor cosmetic results after MxNR, with studies finding 52.6% and 77.6% of patients were dissatisfied with their cosmetic outcomes, respectively [7, 11]. However, there is variation in the literature, with an online survey of a ‘going flat’ support group finding 74.9% of respondents were satisfied [12], although members of such groups may be self‐selected, introducing bias.

Whilst previous studies have investigated the impacts of breast surgery scars, most participants had undergone breast conserving surgery or breast reconstruction. Reid‐de Jong and colleagues [13] investigated the impact of simple mastectomy scars specifically, but only for women who had mastectomy tattooing. Therefore, despite most women not undergoing breast reconstruction post mastectomy, existing research into cosmetic outcomes, satisfaction and quality of life after mastectomy focuses on women who undergo some form of reconstruction, using non‐reconstruction patients simply as a comparison group.

Thusly, little is known about the impact of MxNR scars specifically, as evidenced by the Association of Breast Surgery and James Lind Alliance priority setting partnership who identified the outcomes and impacts of MxNR in two of their ‘Top 10 research priorities for breast cancer surgery’ [14].

This study aimed to explore womens' satisfaction with cosmetic outcomes of MxNR, their attitudes towards their scars and the scars' impacts on their quality of life.

## 2 Methods

### 2.1 Study Design

This was a qualitative semi‐structured interview study with thematic analysis of interview data using the Framework approach. This work has been reported in line with the consolidated criteria for reporting qualitative studies (COREQ) and standards for reporting qualitative research checklist (see Tables S1 and S2).

### 2.2 Ethics and Regulatory Approvals

Research ethics approval was obtained on 03/02/2023 (National Research Ethics Committee; IRAS project ID 313240). Local Research and Innovation approvals were also obtained.

### 2.3 Interview Population

Eligible participants were women who had undergone a unilateral or bilateral mastectomy without reconstruction in the preceding 5 years. The study was limited to those who spoke English as there was no translation service available.

### 2.4 Recruitment Strategy

Recruitment took place from January to April 2024. Participants were recruited via stratified sampling from a pilot study questionnaire [15], where they had previously given consent to be approached for an interview on this topic, or by convenience sampling from routine follow‐up appointment clinics at a single UK Teaching Hospital. Patients were approached during their appointment by the primary researcher and verbally informed about the study. If interested, they were given the study pack.

### 2.5 Topic Guide Development

Interviews were carried out by JM or LL using a topic guide (see Supporting Information S1). Content validity for the topic guide was developed by a literature review and from the results of a questionnaire pilot study [15, 16]. Key topics included: cosmetic satisfaction after simple mastectomy, physical impacts of mastectomy scars, attitudes towards mastectomy scars, mastectomy scars' impact on body image and confidence, and scar revision surgery.

### 2.6 Data Collection

Participants received a study pack containing a cover letter, participant information sheet, and consent form prior to the interview. Written, informed consent was obtained from all participants.

Interviews were conducted face‐to‐face, by telephone or by video call according to patient preference.

### 2.7 Data Analysis

Interviews were recorded and transcribed verbatim and thematic analysis was carried out using the 5 step Framework approach: familiarisation, identifying a thematic framework, indexing, charting and mapping and interpretation [17, 18, 19]. This allows identification of key themes, whilst retaining the context of the data and allowing comparison across and between individual cases [17]. The Framework approach is particularly useful in applied research contexts, which is why it was chosen for this study which had specific questions and pre‐defined objectives.

Recruitment ceased when saturation of themes was reached, that is when no new themes were identified in 5 consecutive interviews. To ensure reliability of codes and themes derived, transcripts were triple coded by LL, with 15% of the data being dually coded by another independent researcher (CM) and cross‐checked for consistency.

### 2.8 Research Team and Reflexivity

Interviews were conducted by LL and JM. Prior to the study, LL received training in the conduct of interviews from JM and LW (breast cancer surgeons and researchers with expertise in qualitative research methodology). Participants were made aware of the researchers' clinical background and research interests. Researcher reflexivity was acknowledged as an inherent part of the conduct of the research and interpretation of findings. Further details are provided in Supporting Information S2.

## 3 Results

Forty‐five women were invited to interview. Twenty of these (44.4%) agreed to participate. Reasons for non‐participation included ill health, time constraints and avoiding distress.

Interviews took place face‐to‐face (8/20, 40%), over the phone (10/20, 50%) or by online video call (2/20, 10%), between March and April 2024. Interview duration was between 21 and 83 min (mean length 47.3 min, SD 15.4).

Table 1 shows the patient demographics. Participants were aged between 47 and 91 years old, with a median age of 63.0 (range 44 years). Mastectomies had been performed between 1998 and 2024, with the median number of years since MxNR being 3.0 (range 1–21 years). Five women (25.0%) had bilateral mastectomies, of whom two were for bilateral cancer (2/5), one for risk‐reduction (1/5) and two for symmetry. Two women (10.0%) had undergone scar revision surgery, with another woman having it scheduled.

**TABLE 1:** Patient and treatment characteristics of interview participants.

Characteristic		Count (%)
Relationship status	Single	2 (10.0)
	Married	14 (70.0)
	Long term partner	1 (5.0)
	Widowed	3 (15.0)
Higher education	Yes	6 (30.0)
	No	14 (70.0)
Sexual orientation	Heterosexual	18 (90.0)
	Lesbian	2 (10.0)
Single or double mastectomy	Single	15 (75.0)
	Double	5 (25.0)
Adverse event	Yes	12 (75.0)
	No	4 (25.0)

**FIGURE 1:**
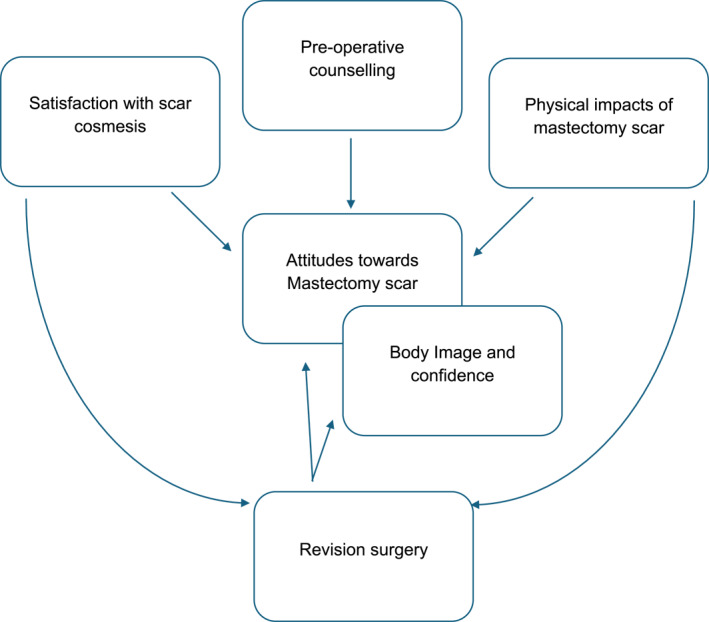
Diagrammatic representation of interactions between themes.

Six major themes were identified: satisfaction with scar cosmesis, physical impacts of scars, attitude towards flat mastectomy scars, body image and confidence, pre‐operative counselling and scar revision surgery (Figure 1).

### 3.1 Theme 1: Satisfaction With Scar Cosmesis

Most women described their MxNR as having poor cosmetic features, including an uneven chest wall, dog ears and high scar placement.

It has a ridge in it, it does come up… Apart from that it’s scarred with the radiotherapy.. I’ve go, too much here, it’s tucked in because it’s a bit err floppy there.

[P4]

It’s quite lumpy bumpy… And at the end I have got… a small dog ear, but that bugs me more than anything.

[P20]

**FIGURE 2:**
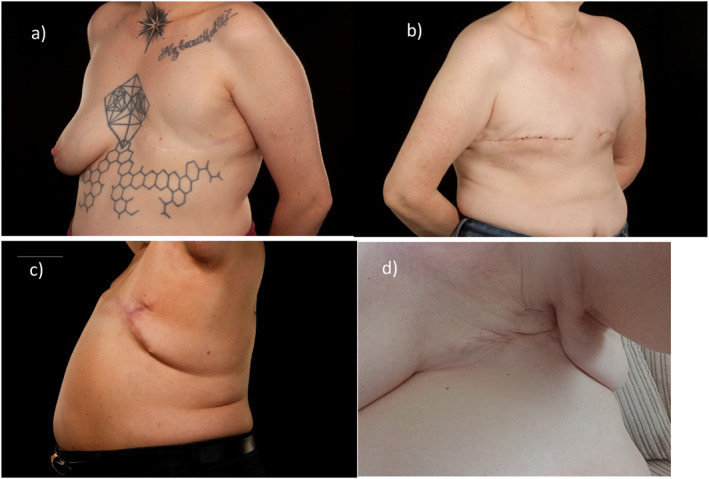
Different types of MxNR; (a) shows a unilateral left‐sided MxNR utilising a U‐shaped curved scar to minimise dog‐ear formation; (b) shows a bilateral MxNR utilising a elliptical incision to give a straight scar; (c) shows a fish‐tail plasty revision of a lateral MxNR scar used to excise a large dog‐ear in a patient with high body mass index; (d) shows an example of a left sided MxNR with tethering and lateral excess.

Other features women disliked included radiotherapy‐induced skin changes; *‘red blotches’, ‘like a bruise’, ‘scaly’*. Few described their scars as *‘flat’*, *‘neat’* or *‘straight across’*, features typically associated with a good cosmetic outcome (Figure 2).

I'm just completely flat. I mean, I’ve got no lumps, bumps… they’re really good scars.

[P1]

Satisfaction with cosmesis aligned with descriptions. Women who described stereotypically good scars were satisfied, whereas those describing suboptimal features were unsatisfied, with two describing their scars as *‘ugly’*.

### 3.2 Theme 2: Physical Impacts of Scars

Women described many physical impacts of their MxNR, including pain, tingling, numbness, or phantom sensations of the breast still being there.

it feels like somebody's got a wire across your chest.

[P1]

All caused distress and served as intrusive reminders of their cancer experience. Tight mastectomy scars restricted arm or shoulder movement, with some reporting an inability to perform activities such as vacuuming, reaching shelves and playing with grandchildren.

playing with my grandson… he's always, always sticking his elbow… into the one place on my chest that actually still hurts.

[P13]

Several women experienced discomfort when wearing bras or carrying out daily activities due to rubbing of lateral excess tissue. For one woman chafing of her lateral excess tissue interfered with her job.

they left what they call a dog ear… being a butcher… I'm moving my arm constantly, it was rubbing.

[P16]

Another woman struggled getting prosthetics to fit properly because of an uneven contoured chest wall.

it went in a lot on one side, it was hard to, well the prosthesis was different, I had one that had like a big tail going round so it could fill that area better.

[P19]

### 3.3 Theme 3: Attitude Towards Mastectomy Scar

Women's attitudes and emotional responses to their scars varied substantially. Depression because of MxNR or its aesthetic impact was only mentioned by a minority. Many viewed their scars as a necessity, expressing a resigned attitude. Older women tended to view their scars more neutrally compared to younger women

as old as I am, I’m not really fussed.

[P8]

Two women who had their first mastectomies 17 and 21 years ago, found that they had become less upset and affected by their scars over time.

well it's been such a long time I don’t really bother about it.

[P4]

Several participants described their scars as a reminder of their cancer and lost breast, conjuring difficult emotions and painful memories, triggering flashbacks for one woman.

It’s just a horrible reminder, and I think I were expecting it to be less of a reminder.

[P10]

Some were upset over missing part of themselves or associated the loss of their breast with a loss of identify or femininity.

But I've always been known for my red hair, and my boobs, me cleavage… it's part of being a female as well.

[P12]

Some felt disfigured and used strong, emotive language to describe their scars, with one stating it looked like her breast *‘had been bitten off by a shark’* and that she had been *‘cut into with a butter knife’* [P10].

Despite being unhappy with their scars, some women attempted to positively reframe their attitudes towards their MxNR, consciously expressing gratitude for being alive and no longer having cancer.

I will always look at my body and think oh dear… I’ve lost both of them. But I’m here, and that’s all there is.

[P2]

Conversely, a few women had positive attitudes towards their MxNR. This was particularly true of the women who had chosen to undergo a contralateral symmetrising or risk‐reducing mastectomy. They voiced improved body image following double mastectomy, as they had either never enjoyed having large breasts or never felt a need for breasts and preferred the symmetry of being completely flat;

I'm more comfortable because I haven't got the, the big boobs. I am a lot more comfortable in my skin now than I was before.

[P1]

One woman, who had a unilateral mastectomy, stated that her scar made her feel proud to have overcome cancer.

that scar is a reminder of how strong I had to be and what I’ve gone through.

[P20]

An emergent theme was the effect of women's sexuality on their attitude towards their MxNR. Two women interviewed identified as lesbian and referenced their sexuality when explaining their attitudes towards their mastectomy scars. One woman expressed an apathetic, and the other a positive attitude. They both stated that they felt their breasts were less important to them due to their identity as gay women.

I'm a gay woman… It doesn't bother me one iota.

[P16]

### 3.4 Theme 4: Body Image and Confidence

Most women reported their MxNR had negatively impacted their body image and confidence, although a few stated it had not. Common concerns included asymmetry and worrying that other people could tell they'd had a mastectomy, feeling self‐conscious about people seeing their scars, being flat chested accentuating their stomach and not feeling feminine.

you think well, will people notice.

[P5]

Consequently, women altered the type of clothing they wore, avoiding tops that would reveal their scars or accentuate their missing breast. Some described not knowing how to dress for their new body shape. A minority felt too self‐conscious to go to social occasions or struggled to go to work after their surgery.

I have absolutely not known what to wear. So that has stopped me going out with my girls and definitely knocked my confidence back.

[P12]

Conversely, one woman who had originally had very large breasts opted to undergo a contralateral symmetrising mastectomy, stated not having big breasts had improved her body image.

Most women wore a prosthesis in their bra when in public, with one stating, *‘I should die if I'd have company and I were sat here, you know, and I'd got nothing on that side’* [P11]. Women also expressed more discomfort in situations where it was harder to hide their MxNR, such as at the gym or when swimming.

Some women avoided looking at their scars in the mirror completely to minimise distress. However, one woman said that she used looking in the mirror to reassure herself that others couldn't tell she'd had a mastectomy. For a minority, discomfort over their scars extended to their partners, with women in long‐term relationships reporting feeling self‐conscious of their partners viewing their scars post‐surgery. However, most expressed that this had decreased over time with partner support and encouragement. Several women stated they were relieved to have long‐term partners, as they felt that they would be embarrassed about their MxNR if they were single.

not everybody's got the support of a husband like mine, and I understand, God if I were single or ought like that I’d be mortified I think.

[P20]

One single woman described how losing her breast made her feel less attractive to potential partners.

you know, being a single person, how am I gonna attract?.

[P12]

### 3.5 Theme 5: Pre‐Operative Counselling

Only one woman interviewed was shown photographs of outcomes of MxNR before her surgery, which she found useful for understanding the body shape she would have post‐surgery.

Most stated seeing photos of MxNR scars would have been beneficial to reduce the initial shock at their post‐surgical appearance, being able to see different scar types, and initiating conversations about scar placement with their surgeon.

Well my mum had breast cancer and a mastectomy on the right hand side …but her scar, it's very very neat and tidy. So I didn't really. I suppose it would have really helped me if I'd have been able to see somebody who looks like I do now. Cause I'm not tidy, if you know what I mean?.

[P13]

Conversely, some women didn't think seeing photos would be beneficial, as they had already made their decision, or thought they might scare people. Others felt that photos could never prepare them for the shock and grief they felt when they first saw their chest post‐MxNR.

Some women described being negatively affected by media portrayals of MxNR, stating they are not representative. These women tended to have scars with poor cosmetic features and so compared their own scar to those shown on television and were consequently upset that their scars were not flat, faint and *‘perfect’* like those shown in the media.

Mine looks nothing like some of the ladies.

[PX]

A Macmillan TV advert was frequently mentioned, with two women, who both had poor cosmetic outcomes, disliking it. Conversely, one woman, who had a good cosmetic outcome, thought it was *‘brilliant’* [P11]. A woman who ran the London marathon topless after having a double MxNR was mentioned by several women, one describing her as *‘brave’* and important for raising awareness of those who don't have reconstruction, whilst another woman felt upset as she compared her own uneven scars to the runner's flat ones.

And I thought ‘oh why can't I look like that?’ I want to be like that’. She was flat chested.

[P18]

### 3.6 Theme 6: Scar Revision Surgery

When asked how they would improve their scars, women responded they would prefer scars that were flat, straight across and low on the chest wall, without dog ears. Despite this, less than half had discussed scar revision surgery with their breast surgeon and some weren't aware it was an option.

Those who underwent scar revision surgery were highly satisfied with the results. One woman had scar revision surgery to remove a lump and lateral dog ear. She expressed satisfaction with the outcome, stating that she thought her new fishtail scar was *‘tidier’* than a dog ear.

Two women requested scar revision surgery for lateral dog ears but were refused by their surgeon due to previous significant post‐operative complications increasing the risk for poor healing, and local cancer recurrence respectively. The remaining two women who discussed scar correction with their surgeon decided against it as they didn't want further surgery.

I've had enough done to me and I just want to get used to being the new me and if that's part of the new me, then that's part of me.

[P2]

Some women were interested in tattoos to improve the cosmesis of their MxNR, but none had undergone tattooing at the time of interviewing.

I'm gonna get tattoos to cover the scarring. Just so it's not there every time you're… looking the mirror or anything and just makes it look a bit… better… Just something a bit feminine.

[P9]

## 4 Discussion

This study explored cosmetic outcomes of MxNR in women from a single NHS trust. This area of research has long been neglected, with a heavy focus on women who have reconstruction in the published literature, even though most patients undergoing mastectomy in the UK do not have reconstruction surgery. Most participants expressed an attitude of neutrality and acceptance towards their MxNR, viewing the result as essential to overcoming cancer; these women were often unsatisfied with, but not upset by, their scar's cosmetic appearance. Participants confirmed what is known regarding the negative impacts a MxNR can have, using harsh, emotive descriptions, replicating those from previous studies where women described feelings of damage and mutilation [13, 20, 21]. This study found that women who identified as lesbian seemed less affected by the loss of their breasts due to their sexuality, although no inferences can be generalised based on a sample size of two. However, this does align with the existing literature, with lesbian women stating that their sexuality contributed to their decision to live flat [22]. Rubin and Tanenbaum [23] suggest that lesbian women have already challenged society's heteronormative ideas of gender, femininity and the male gaze through their sexual orientation, and so may find it easier to separate their breasts from their personal gender identity and live flat.

The prevalence of poor scar quality was high but in line with previous studies [7, 11]. However, a key theme identified was the correlation between dissatisfaction with scar cosmesis and the distress these poor outcomes caused, particularly those left with dog‐ears or uneven chest wall contour, which worsens these impacts further. It is imperative that a flat chest wall contour is achieved to facilitate the use of breast prosthesis post‐MxNR to prevent the prosthesis dislodging or falling out of their bra, causing embarrassment [24, 25]. Breast prosthetics have been shown to improve body‐image and sense of femininity, giving patients more confidence to go out in public [24, 25].

This study identified a need for revision surgery to achieve aesthetic flat closure for some patients for similar reasons to those found by Evaraas and colleagues [21]. Studies [7, 26] have shown that scar revision surgery improves women's emotional, physical and psychosocial quality of life and out findings support this. Scar revision techniques may include excision of scars or excess tissue or the use of lipo‐modelling to achieve a smoother, more even chest wall [8, 27, 28]. However, it is imperative that the cosmetic aspects of MxNR are considered to optimise outcomes for patients, utilising Aesthetic Flat Closure techniques at the index surgery in the interest of ‘Getting It Right First Time’ [3]. In particular, the attitudes held by surgeons that lateral adiposity is not breast tissue and therefore doesn't need excising at MxNR surgery [29] needs to be challenged, as even small dog‐ears impacted greatly on patient satisfaction in this study.

There are several suggested techniques in the literature for improving primary cosmetic outcomes of MxNR to achieve aesthetic flat closure. These include pre‐operatively marking women while they are seated upright so that the distribution of soft tissue can be assessed, women can also be seated upright intraoperatively with provisional staple closure performed to assess whether flat closure has been achieved [8, 30]. This allows lateral excess skin to be removed, preventing ‘dog ear’ formation. For women with high body mass indices, dog ears present a more significant challenge, however there are techniques for preventing these, including ‘lateral de‐fatting’ to facilitate a smooth contour, extension of the incision laterally or the use of fish‐tail or Fleur di Lis (anchor) incisions [8, 30]. Furthermore, ensuring consistent flap thickness is essential to achieve a symmetrical, smooth chest wall [8, 30] and surgeons should take care to develop their surgical technique and train resident doctors so that they are able to provide cosmetically pleasing outcomes.

Asymmetry was one of the most concerning issues for women in this study and several were interested in bilateral mastectomy to improve this. Contralateral symmetrising mastectomy is an option growing numbers of women with unilateral breast cancer are choosing [31]. Women who chose to ‘go flat’ by undergoing contralateral symmetrising surgery generally have high rates of satisfaction [31] and the study by Tyner and colleagues [7] found women felt liberated and empowered after MxNR by being allowed to make their own choice to live flat. In view of this, and in line with NHS priorities to facilitate Shared Decision Making [32, 33], CSM should be offered as an alternative to breast reconstruction for women who are unable to have or do not want to undergo a reconstruction but wish for better symmetry.

A recurring theme was that some women struggled to look at their MxNR scars in the mirror, consistent with studies investigating women's initial mirror‐viewing experiences [7, 13, 34]. A lack of preparedness may contribute to this and could potentially be mitigated by showing photographs of MxNR outcomes pre‐operatively during informed consent. Showing photographs of diverse outcomes may also reduce distress caused by women's comparison of their MxNR with those in the media and aligns with research in the reconstruction‐setting where women wanted to be shown a range of photographs depicting different outcomes when making decisions about breast reconstruction [35]. A previous study has highlighted that healthcare professionals don't routinely show photographs of MxNR to patients, despite consensus that it would be advantageous [29], and this study confirms these findings. Current breast surgery UK guidelines [36] recommend photographs are shown to patients having mastectomy who are considering breast reconstruction pre‐operatively as part of the informed consent process but there are no similar recommendations for those patients having MxNR. A national audit demonstrated this disparity, with only 25% of MxNR patients shown photographs during the consent process compared to 80% of reconstruction patients [37]. Ketley's and colleagues' study of breast clinicians found this discrepancy was mainly due to lack of time in clinic for patients undergoing MxNR compared to breast reconstruction [29]. Evidence suggests patients are inadequately counselled before their MxNR, with one study finding only 43% patients felt satisfied with the information provided preoperatively as to what their scar would look like [36]. There appears to be gap in patient counselling between reconstruction patients and MxNR patients, as most patients undergoing reconstruction are routinely allocated more time to show reconstruction photos during reconstruction clinics. MxNR patients experience similar levels of quality‐of‐life effects as MxR patients [38] so there may be a need to re‐evaluate if elements of MxR counselling should be applied to MxNR. For many women having mastectomy, breast conservation is not an option and they do not have a choice about keeping their breast, so clinicians may perceive there is less need for preoperative counselling. However, our data suggest that many women are unprepared for the outcome of the MxNR surgery, so it is clear that more preoperative input is needed.

There is a concern that some women may find pictures of suboptimal cosmetic outcomes frightening, which is consistent with a study by Herring and colleagues [39]. Thusly, surgeons need time to have a detailed, nuanced conversation with patients, in which they show them a range of photos of different outcomes, whilst explaining the likelihood of such outcomes and the measures they would take to avoid them. This would provide the patient with the information they desire whilst alleviating their fears. Surgeons also need to make more effort to give these women a good aesthetic flat closure in the first place.

Similarly, women who have reconstruction often have several follow‐up visits with their surgeons to optimise cosmesis, whereas MxNR patients may be less likely to be seen by their surgeons and if they are, often no focus is given to scar cosmesis. The patient pathway needs improving to include more time for surgical consultation to discuss potential outcomes from MxNR, discuss risk factors and consider incision type and placement. This is increasingly important as studies also indicate that women choosing MxNR often report feeling pressured towards reconstruction and under‐informed about flat outcomes [7]. Shared decision‐making in this area is therefore imperative, and clinicians should be tailoring their consultations to the flat option, using visual aids and outcome photographs where appropriate. Intra‐operatively they should using the necessary skills and techniques to achieve aesthetic flat closure after MxNR and be aware of the outcomes that patients prioritise (such as absence of dog ears), using revision surgery to correct these if adequate cosmetic outcomes are not achieved.

### 4.1 Implications (Clinical and Research)

This paper contributes to the under‐researched topic of aesthetic outcomes of MxNR. More research is needed to understand and compare the outcomes for patients having MxNR, particularly with regards to aesthetic flat closure. However there is an urgent need to address the disparity in the patient pathways between patients undergoing MxNR with their counterparts undergoing reconstruction. Equivalent clinical time should be allocated to every patient to allow a full and comprehensive discussion of the potential outcomes and to make a surgical plan, with a focus of incision selection and any special techniques required to remove lateral excess.

### 4.2 Limitations

Limitations of this study include the risk of self‐selection bias, meaning women who had particularly negative experiences may not have wanted to participate due to finding the topics upsetting, or alternatively, women who had poor outcomes volunteering as they wished to express their negative experience. Furthermore, the use of convenience sampling could lead to selection bias. For example, most interview participants were recruited from the breast prosthetics clinic, meaning women who don't wear prostheses may be underrepresented. The use of semi‐structured interviews allowed for deep exploration of women's experiences and feelings, with the opportunity to explore emergent themes. Interviewer bias must be acknowledged as a risk as most interviews were carried out and coded by the same interviewer which could potentially result in missed themes. Furthermore, the nature of qualitative interviews means these results may be subject to recall bias.

This study is a single‐centre study, which limits the generalisability of the results. Additionally the study did not specifically collect data on socio‐economic status or ethnicity but participants were all white female English speakers, further limiting the generalisability of these findings.

Interpretation of the results was performed with patient advocates from the charity Flat Friends UK, however the data collection and analysis were performed independently to minimise the potential for advocacy bias.

## 5 Conclusion

MxNR can have a profound impact on quality of life. It is therefore of utmost important that the MxNR is performed in a way to achieve the optimal outcome for each individual to minimise the effects of a poor outcome compounding these issues. Breast clinicians need to familiarise themselves with all MxNR techniques and allow more clinic time for patients undergoing MxNR. This will facilitate proper shared decision making, improved pre‐operative counselling and enable post‐operative review. Consequently, these changes would allow optimisation of cosmetic outcomes, allow revision surgery to be arranged where needed and bring the pathway for MxNR in line with that of breast reconstruction, reducing the disparity between these two groups of patients.

## Author Contributions

**Laura Lyons:** data curation, formal analysis, investigation, methodology, project administration, resources, writing – original draft. **Charlene Martin:** formal analysis, methodology, project administration, supervision, validation, writing – review and editing. **Lynda Wyld:** conceptualization, formal analysis, investigation, methodology, project administration, resources, supervision, validation, writing – review and editing. **Nicola King:** formal analysis, writing – review and editing. **Sam Brunsden:** formal analysis, writing – review and editing. **Jenna Morgan:** conceptualization, data curation, formal analysis, investigation, methodology, project administration, resources, supervision, validation, visualization, writing – original draft, writing – review and editing.

## Funding

The authors have nothing to report.

## Ethics Statement

Research ethics approval was obtained on 03/02/2023 (National Research Ethics Committee REC reference 22/PR/1455; IRAS project ID 313240). Local Research and Innovation approvals were also obtained on 19^th^ December 2022 (DBTH reference: 1197/2023/NCTS).

## Consent

Human subjects were involved. All patients gave written informed consent prior to recruitment to the study. I have stated that Informed Consent was obtained, or provided an explanation in my Methods section.

## Conflicts of Interest

The authors declare no conflicts of interest.

## Supporting information


Supporting Information S1

Supporting Information S2

**Table S1:** Consolidated criteria for reporting qualitative studies (COREQ) checklist.

**Table S2:** Standards for Reporting Qualitative Research (SRQR) checklist.

## Data Availability

The data that support the findings of this study are available on request from the corresponding author. The data are not publicly available due to privacy or ethical restrictions.
