# Supplementary material for: Insights Into ‘Living Flat’: A Qualitative Study of Patients Who Have Mastectomy Without Reconstruction
Source: Psychooncology. 2026 Mar 26;35(4):e70436. doi: 10.1002/pon.70436 (PMC13021570; doi:10.1002/pon.70436)
Supplement: Supplementary file 1 — Supporting Information S1 [file PON-35-e70436-s001.docx]

***Supplementary File 1: Interview Schedule***

**Interview Topic Guide**

**(Health Care Professionals)**

**(Version 1)**

| Name of researcher: |
| --- |
| Date of interview: |
| Participant ID: |

**Welcome and introductions:**

- Thank the patient for giving up their time to speak with us
- Reiterate the aims of the study
- Explain how the interview will work
  - Length and format
  - How it will be recorded
  - Reassure that you can repeat or clarify questions
  - Reassure that there are no right or wrong answers
  - You can take a break or stop at any time

**Before we start the interview, can I ask if you have:**

- Read the information sheet?
- Signed the consent form?
- Any questions before we begin?

**Background and history**This section will cover background and medical history:

- How long ago was your breast surgery?
- Can you take me through the type of surgery you had *(prompts: unilateral or bilateral, did you need to undergo any additional surgeries)*
- Did you have any other treatments alongside/after your surgery? *(chemotherapy, radiotherapy, hormone therapies)*

**Decision making**This section will cover decision-making, information and discussions:

- Thinking back to when you were making your treatment decision, can you remember any of the information you looked at? *(prompts: leaflets, websites).*
- Did the surgeon show you any photographs or examples? *(prompt: if yes, did this prepare you for what to expect?)*
- Did you discuss the decision with anyone else? *(prompts: friends, family, breast nurses, consultant at the breast clinic?).*
- How long did you take to make your decision?

**Satisfaction with surgery results**This section will cover satisfaction with surgery:

- Were you satisfied with the results? Exploring factors such as:
  - Expectations of surgery
  - Size, shape and symmetry involved in the whole appearance and feel
  - Posture
  - How comfortably does your clothing sit (on top)

**Physical well-being**

- Following your surgery, did you have any complications or side effects?
- Did the recovery period have an impact on your daily activities? (short/long-term?)
- Do you experience any pain in the muscles of your chest or pain from the scar?
- How long did it take to recover/for scar healing?

**Satisfaction with the hospital and care team**

- Do you feel you had enough access to support and information while making your decision?
- Did you feel you had enough information about the risks involved and what to expect?
- Did you have enough time to make your decision and research the surgical options?
- Did you receive any additional counselling?

**Reconstruction**

- Why did you decide not to have immediate reconstruction? *(prompt: if surgery was during the pandemic, did you face a delay in waiting lists?)*
- Are you planning to have reconstruction in the future?
- Are you happy with your decision? If not, why?

**Demographics**

- Age
- Marital status
- Level of education
- Occupation

**Wrap up**

- Is there anything else you would like to tell us about your experience that we have not covered in this interview?
- Thank the participant for taking part in the interview
