# Supplementary material for: Insights Into ‘Living Flat’: A Qualitative Study of Patients Who Have Mastectomy Without Reconstruction
Source: Psychooncology. 2026 Mar 26;35(4):e70436. doi: 10.1002/pon.70436 (PMC13021570; doi:10.1002/pon.70436)
Supplement: Supplementary file 2 — Supporting Information S2 [file PON-35-e70436-s002.docx]

**Supplemental File 2: Reflexivity statement:**

While conducting this research we acknowledge that our personal backgrounds,

experiences and own bias which have influenced the research analysis and

interpretation of the outcomes. The primary interviewer and analyst is a young female

with a medical background. She may have approached the research with a particular

lens which could have influenced the questions she asked in participant interviews and

the way she analysed the results. We understand that her background and assumptions

about the participants opinions may have led to limitations and benefits within the study.

Throughout the research period she attempted to keep a critical awareness of how her

perspectives and experiences could influence the outcomes of the research. By

maintaining a reflective mind-set, she aimed to keep the findings grounded in data rather

then her own bias.
