# Supplementary material for: Insights Into ‘Living Flat’: A Qualitative Study of Patients Who Have Mastectomy Without Reconstruction
Source: Psychooncology. 2026 Mar 26;35(4):e70436. doi: 10.1002/pon.70436 (PMC13021570; doi:10.1002/pon.70436)
Supplement: Supplementary file 3 — Table S1: Consolidated criteria for reporting qualitative studies (COREQ) checklist. [file PON-35-e70436-s004.docx]

**Supplementary Table 1:** ***Consolidated criteria for reporting qualitative studies (COREQ) checklist***

| **No. Item** | **Guide questions/description** | **Reported** |
| --- | --- | --- |
| **Domain 1: Research team and reﬂexivity** | | See reflexivity statement |
| *Personal Characteristics* | | |
| 1. Interviewer/facilitator | Which author/s conducted the interviews? | LL, JM |
| 2. Credentials | What were the researcher’s credentials? | JM: FRCS, PhD, DipMedEd, MBChB |
| 3. Occupation | What was their occupation at the time of the study? | JM: Academic oncoplastic surgeon and NIHR Advanced Fellow  LL: medical student, BSc research student |
| 4. Gender | Was the researcher male or female? | Female |
| 5. Experience and training | What experience or training did the researcher have? | JM has qualitative research experience across >10 studies using both interviews and focus groups |
| *Relationship with participants* | | |
| 6. Relationship established | Was a relationship established prior to study commencement? | For some participants |
| 7. Participant knowledge of the interviewer | What did the participants know about the researcher? e.g. personal goals, reasons for doing the research | Role and purpose made explicit |
| 8. Interviewer characteristics | What characteristics were reported about the interviewer/facilitator? e.g. Bias, assumptions, reasons and interests in the research topic | Research interests stated in methods, biases stated in limitations section |
| **Domain 2: study design** | |  |
| *Theoretical framework* | | |
| 9. Methodological orientation and theory | What methodological orientation was stated to underpin the study? | Thematic analysis |
| *Participant selection* | | |
| 10. Sampling | How were participants selected? e.g. purposive, convenience, consecutive, snowball | Convenience |
| 11. Method of approach | How were participants approached? e.g. face-to-face, telephone, mail, email | Face-to-face |
| 12. Sample size | How many participants were in the study? | 20 |
| 13. Non-participation | How many people refused to participate or dropped out? Reasons? | No drop-outs, 25 declined to take part due to ill health, time constraints and avoiding distress |
| *Setting* | | |
| 14. Setting of data collection | Where was the data collected? e.g. home, clinic, workplace | Workplace, online, by telephone |
| 15. Presence of non-participants | Was anyone else present besides the participants and researchers? | No |
| 16. Description of sample | What are the important characteristics of the sample? e.g. demographic data, date | Demographics provided (Table 1) |
| *Data collection* |  |  |
| 17. Interview guide | Were questions, prompts, guides provided by the authors? Was it pilot tested? | Interview topic guide included in supplemental materials |
| 18. Repeat interviews | Were repeat interviews carried out? If yes, how many? | No |
| 19. Audio/visual recording | Did the research use audio or visual recording to collect the data? | Yes |
| 20. Field notes | Were ﬁeld notes made during and/or after the interview or focus group? | Yes |
| 21. Duration | What was the duration of the interviews or focus group? | Ranged between 21 and 83 minutes (mean length 47.3 minutes) |
| 22. Data saturation | Was data saturation discussed? | Yes |
| 23. Transcripts returned | Were transcripts returned to participants for comment and/or correction? | No |
| **Domain 3: analysis and ﬁndings** | |  |
| *Data analysis* | | |
| 24. Number of coders | How many data coders coded the data? | 2 |
| 25. Description of coding | Did authors provide a description of the coding tree? | No, may be requested |
| 26. Derivation of themes | Were themes identiﬁed in advance or derived? | Both |
| 27. Software | What software was used to manage the data? | NVivo and Microsoft® Word |
| 28. Participant checking | Did participants provide feedback on the ﬁndings? | No |
| *Reporting* | | |
| 29. Quotations presented | Were participant quotations presented to illustrate the themes/ﬁndings? Was each quotation identiﬁed? | Yes |
| 30. Data and ﬁndings consistent | Was there consistency between the data presented and the ﬁndings? | Yes |
| 31. Clarity of major themes | Were major themes clearly presented in the ﬁndings? | Yes, in results |
| 32. Clarity of minor themes | Is there a description of diverse cases or discussion of minor themes? | Yes, in discussion |
